# Evaluation of the clinical efficacy of endoscopic endonasal dacryocystorhinostomy in treating chronic dacryocystitis

**DOI:** 10.3389/fmed.2026.1868878

**Published:** 2026-09-30

**Authors:** Zhang Cai, Jingfeng Wang, Zuhong Chen, Dongdong Huang, Lei Cai

**Affiliations:** 1 Otorhinolaryngology Department, The First Hospital of Putian, Putian, China; 2 Ophthalmology Department, The First Hospital of Putian, Putian, China

**Keywords:** chronic, clinical treatment, efficacy, dacryocystitis, endonasal, endoscopic

## Abstract

**Objective:**

To compare the short-term clinical efficacy and safety of endoscopic endonasal dacryocystorhinostomy (DCR) and traditional external DCR in patients with chronic dacryocystitis.

**Methods:**

A retrospective analysis was conducted on the clinical records of patients with chronic dacryocystitis treated at our hospital from November 2022 to November 2025. Patients were retrospectively categorized according to the surgical procedure received: the research group underwent endoscopic endonasal DCR, whereas the control group underwent traditional external DCR. Clinical data from all patients were collected and analyzed.

**Results:**

No statistically significant differences were observed in the recorded baseline characteristics between the two groups (*P* > 0.05). The clinical efficacy between the two groups showed no significant difference (*P* > 0.05), nor did the main symptom scores (*P* > 0.05). However, the research group demonstrated significantly lower intraoperative blood loss and shorter postoperative hospital stays compared to the control group (*P* < 0.05). The overall incidence of postoperative adverse events was also lower in the research group than in the control group (*P* < 0.05), and self-reported patient satisfaction was higher in the research group than in the control group (*P* < 0.05).

**Conclusion:**

Within the 2-month postoperative follow-up, unadjusted comparisons showed that endoscopic endonasal DCR was associated with lower intraoperative blood loss, shorter postoperative hospitalization, a lower overall incidence of postoperative adverse events, and higher self-reported patient satisfaction than traditional external DCR. No statistically significant between-group differences were detected in short-term anatomical or functional success. Larger prospective studies with adjustment for potential confounders and longer follow-up are required to confirm these findings.

## 1 Introduction

Chronic dacryocystitis is a common ophthalmologic condition characterized primarily by lacrimal duct obstruction and lacrimal sac infection. Patients often present with persistent tearing, ocular discomfort, swelling, and tenderness in the lacrimal sac area. In severe cases, complications such as acute dacryocystitis or orbital cellulitis may occur, potentially jeopardizing visual function and overall quality of life (1–3). Therefore, developing effective treatment strategies for chronic dacryocystitis is of significant clinical importance. Traditionally, the treatment of chronic dacryocystitis has relied on external dacryocystorhinostomy (DCR). This approach involves making a skin incision to access the lacrimal sac and creating a drainage passage between the lacrimal sac and the nasal cavity, which typically achieves a high success rate. However, external DCR is associated with facial scarring, extensive surgical trauma, prolonged recovery times, and a relatively higher rate of adverse events. With advancements in medical technology, minimally invasive surgical techniques have gained widespread acceptance. The introduction of endoscopic techniques offers a new option for the treatment of chronic dacryocystitis. Endoscopic endonasal DCR is a novel surgical method characterized by minimal invasiveness. Under endoscopic guidance, a direct drainage pathway is established between the lacrimal sac and the nasal cavity, eliminating the need for external incisions on the face. In recent years, the clinical application of endoscopic endonasal DCR has increased, demonstrating promising efficacy and safety (4, 5).

Although endoscopic techniques have achieved considerable progress in the treatment of chronic dacryocystitis, further comparative studies on their clinical efficacy and safety relative to traditional external DCR are still needed. This study retrospectively analyzes clinical data from patients treated for chronic dacryocystitis between November 2022 and November 2025, comparing the outcomes of endoscopic endonasal DCR with those of traditional external DCR. The findings aim to provide a reference for optimizing treatment strategies for chronic dacryocystitis.

## 2 Materials and methods

### 2.1 Ethical statement

This retrospective study was approved by the Ethics Committee of The First Hospital of Putian (Approval No.: 2025-035; approval date: 2025-12-25) and was conducted in accordance with the Declaration of Helsinki. The requirement for informed consent was waived by the Ethics Committee due to the retrospective nature of the study.

### 2.2 General information

This retrospective study included patients diagnosed and treated for chronic dacryocystitis at our hospital between November 2022 and November 2025. A total of 40 eligible patients were identified from the medical records and retrospectively categorized according to the surgical procedure actually received. The choice of surgical approach was made during routine clinical care after preoperative assessment of lacrimal drainage anatomy and overall surgical suitability, taking into account the treating surgeon's clinical judgment and the patient's preference after discussion of the characteristics, potential benefits, and risks of both procedures. No random allocation was performed. The research group comprised 20 patients who underwent endoscopic endonasal dacryocystorhinostomy (DCR), whereas the control group comprised 20 patients who underwent traditional external DCR.

### 2.3 Inclusion and exclusion criteria

Inclusion Criteria:

No restriction on gender.
Complete clinical data available.
Diagnosis of chronic dacryocystitis confirmed by clinical examination and imaging findings, with lacrimal drainage obstruction localized to the nasolacrimal duct and no evidence of proximal canalicular or common canalicular obstruction (6, 7).

Exclusion Criteria:

Patients with contraindications for surgery.
Patients with cardiac, pulmonary, hepatic, or renal insufficiency.
Patients with systemic or autoimmune diseases.
Patients with nasal polyps or nasal tumors.
Patients with mental disorders.
Patients in poor overall condition who were only suitable for lacrimal sac excision.
Patients with isolated canalicular or common canalicular obstruction or other proximal lacrimal drainage obstruction not suitable for standard dacryocystorhinostomy.
Patients with coagulation disorders.

### 2.4 Methods

Before surgery, the level of lacrimal drainage obstruction was assessed based on the available ophthalmic examination and imaging findings. Only patients with obstruction localized to the nasolacrimal duct and without proximal canalicular or common canalicular obstruction were included in the comparative analysis.

Control Group: The control group underwent traditional external DCR. Under general or local anesthesia, a skin incision was made along the Lang's line at the medial canthus. The orbicularis oculi muscle was dissected, and the medial canthal ligament was exposed and divided. After exposing the lacrimal sac, dissection was performed along the periosteum. The frontal process of the maxilla, lacrimal bone, and ethmoid bone within the lacrimal sac fossa were removed to create a bony window and expose the nasal mucosa. The lacrimal sac and nasal mucosa were incised in an “H” shape to form anterior and posterior flaps, which were then sutured together. The incision was closed in layers and compression dressing was applied.

Research group: The research group underwent endoscopic endonasal DCR in three steps: After general or local anesthesia, a “C”-shaped mucosal flap was created at the anterior portion of the uncinate process on the lateral nasal wall to expose the lacrimal bone and frontal process of the maxilla. Using bone punches, the frontal process of the maxilla, lacrimal bone, and ethmoid bone were removed to fully expose the lacrimal sac. The medial wall of the lacrimal sac was incised and turned outward. It was sutured to the “C”-shaped mucosal flap using absorbable sutures. No packing or compression dressing was required postoperatively.

### 2.5 Observation indicators

Postoperative Outcome Assessment: Postoperative outcomes were assessed at 2 months after surgery based on the documented outpatient follow-up findings. Anatomical success and functional success were evaluated separately. Anatomical success was defined as a patent lacrimal drainage pathway, demonstrated by free passage of irrigation fluid into the nasal cavity during lacrimal irrigation and a patent rhinostomy without obvious closure on nasal endoscopic examination. Functional success was defined as the absence of persistent troublesome epiphora or purulent reflux during daily activities and no requirement for additional lacrimal intervention during the follow-up period. For continuity with the original clinical evaluation, a composite clinical response was also recorded as a secondary outcome and categorized as excellent, good, or poor. An excellent response was defined as both anatomical and functional success without postoperative complications. A good response was defined as anatomical success accompanied by marked improvement in lacrimal symptoms, with no complications or only mild self-limited complications. A poor response was defined as anatomical failure and/or persistent clinically relevant symptoms requiring further treatment. The overall clinical response rate was calculated as the proportion of patients with an excellent or good response.

Symptom Scores: Key symptoms, including medial canthal discharge, tearing, and tenderness in the lacrimal sac area, were observed and scored at 2 months after surgery based on symptom severity: 1 point—no tearing, no tenderness, no medial canthal discharge; 2 points—mild tenderness, slight medial canthal discharge, occasional tearing; 3 points—moderate tenderness, noticeable medial canthal discharge, frequent tearing; 4 points—severe tenderness, substantial medial canthal discharge, frequent tearing.

Surgery-Related Indicators: Surgical time, intraoperative blood loss, and postoperative hospital stay were compared between the two groups to evaluate surgical process and recovery efficiency.

Adverse Events: Postoperative adverse events occurring during the 2-month follow-up period were identified from the medical records and follow-up documentation and categorized as infection, hemorrhage, or other documented complications. For each category, the number and proportion of affected patients were recorded. The overall adverse-event incidence was calculated as the proportion of patients who experienced at least one postoperative adverse event; each patient was counted only once in the overall incidence regardless of the number of events.

Treatment Satisfaction: Patient satisfaction was assessed at the 2-month postoperative follow-up using a study-specific three-category questionnaire. Patients independently reported their satisfaction as very satisfied, satisfied, or dissatisfied based on their overall perception of the surgical experience, postoperative recovery, symptom improvement, and cosmetic outcome. The overall satisfaction rate was calculated as (very satisfied+satisfied)/total number of patients×100%. This questionnaire was developed for routine clinical evaluation and had not undergone formal psychometric validation. Because satisfaction was based on patient self-report, no independent clinician-rated assessor was used. In addition, blinding of patients to the surgical approach was not feasible because the external and endoscopic procedures were visibly distinguishable.

### 2.6 Data analysis

GraphPad Prism 8 was used for graphical presentation, and SPSS 26.0 was used for statistical analysis. The normality of continuous variables was assessed using the Shapiro–Wilk test. Normally distributed continuous variables were expressed as mean ± standard deviation (SD) and compared between groups using the independent-samples t-test. Non-normally distributed continuous variables were expressed as median (interquartile range, IQR) and compared using the Mann–Whitney U test. Because the symptom scores were measured on a 4-point ordinal scale, they were treated as ordinal variables and compared between groups using the Mann–Whitney U test rather than parametric tests. Categorical variables were expressed as *n* (%) and compared using Pearson's chi-square test when the assumptions for this test were satisfied; Fisher's exact test was used when expected cell counts were small. Exact two-sided *P* values and 95% confidence intervals (CIs) were reported where applicable. Statistical significance was defined as *P* < 0.05. Given the small sample size and limited number of outcome events, no multivariable regression or propensity-score adjustment was performed. Accordingly, all between-group comparisons should be interpreted as unadjusted exploratory analyses rather than estimates of independent treatment effects. No *a priori* sample size or power calculation was performed because this was a retrospective analysis based on the eligible cases available during the predefined study period. The study was not designed as an equivalence or non-inferiority study.

## 3 Results

### 3.1 Baseline characteristics

In the research group (*n* = 20), there were 8 males and 12 females. The patients were aged 33–65 years, with a mean age of 50.17 ± 8.21 years, and the disease duration ranged from 0.6 to 3.9 years, with a mean duration of 2.18 ± 0.91 years. In the control group (*n* = 20), there were 7 males and 13 females. The patients were aged 32–64 years, with a mean age of 50.54 ± 7.86 years, and the disease duration ranged from 0.5 to 4.0 years, with a mean duration of 2.25 ± 0.88 years. The values following “±” represent standard deviations (SDs). No statistically significant differences were observed in the recorded baseline characteristics between the two groups (*P* > 0.05), as shown in Table 1.

**Table 1:** Comparison of baseline characteristics between the two groups.

	Gender	Research group	Control group	*t*	*P*
Number of Cases	–	20	20	–	–
Gender	Female	12	13	–	–
	Male	8	7	–	–
Age	–	33–65	32–64	–	–
	Mean	50.17 ± 8.21	50.54 ± 7.86	0.146	0.885
Course of disease	–	0.6–3.9	0.5–4.0	–	–
	Mean	2.18 ± 0.91	2.25 ± 0.88	0.247	0.806

Continuous variables are presented as mean ± standard deviation (SD) unless otherwise indicated. The values following “±” are SDs rather than standard errors.

### 3.2 Clinical efficacy

At the 2-month postoperative assessment, anatomical success was achieved in 18 of 20 patients (90.0%) in the research group and 17 of 20 patients (85.0%) in the control group, with no statistically significant between-group difference (Fisher's exact *P* = 1.000). Functional success was observed in 18 of 20 patients (90.0%) in the research group and 16 of 20 patients (80.0%) in the control group, and the difference was also not statistically significant (Fisher's exact *P* = 0.661). For the secondary composite clinical response, 6 patients (30.0%) in the research group had an excellent response, 12 (60.0%) had a good response, and 2 (10.0%) had a poor response. In the control group, the corresponding numbers were 6 (30.0%), 10 (50.0%), and 4 (20.0%), respectively. The overall clinical response rate was 90.0% (18/20) in the research group and 80.0% (16/20) in the control group. No statistically significant difference in the overall clinical response rate was detected between the groups (Fisher's exact *P* = 0.661; OR = 2.25, 95% CI: 0.36–13.97), as shown in Figure 1.

**Figure 1:**
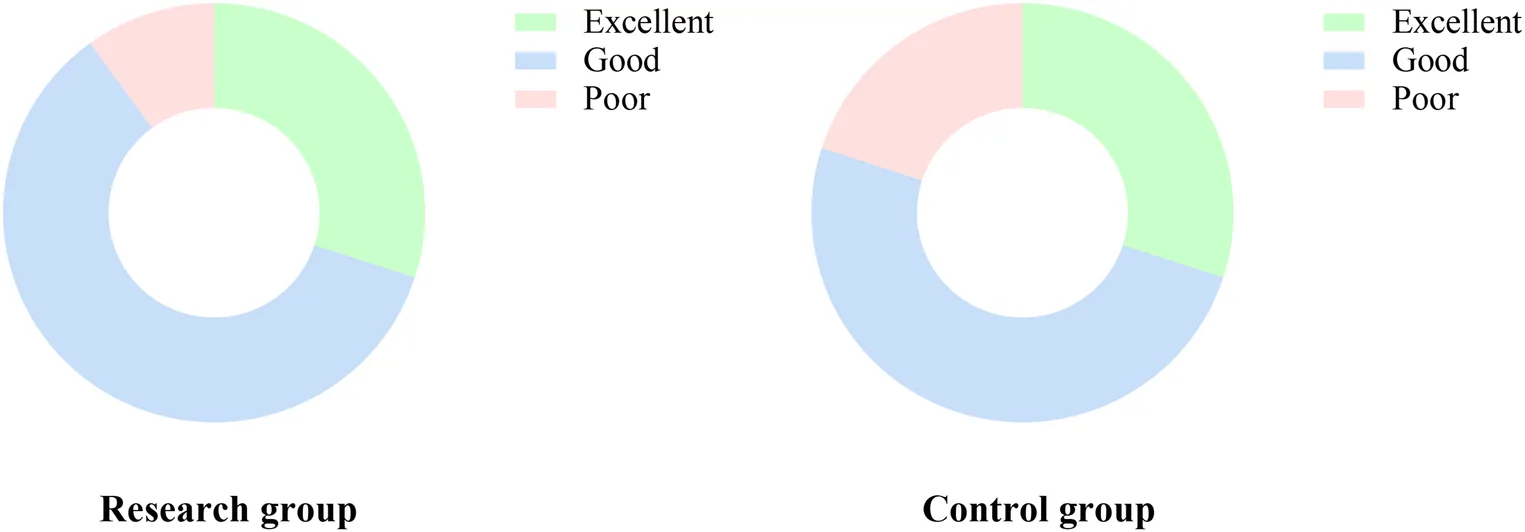
Comparison of composite clinical response between the two groups.

### 3.3 Symptom assessment

At 2 months after surgery, the medial canthal discharge score was 1.0 (IQR, 1.0–1.0) in the research group and 1.0 (IQR, 1.0–2.0) in the control group (Mann–Whitney U = 180.0, *P* = 0.482). The tearing scores were 1.0 (IQR, 1.0–2.0) and 1.0 (IQR, 1.0–2.0), respectively (U = 190.0, *P* = 0.759), while the lacrimal sac tenderness scores were 1.0 (IQR, 1.0–1.0) and 1.0 (IQR, 1.0–1.0), respectively (U = 190.0, *P* = 0.696). No statistically significant between-group differences were observed in any of the three ordinal symptom scores, as shown in Figure 2.

**Figure 2:**
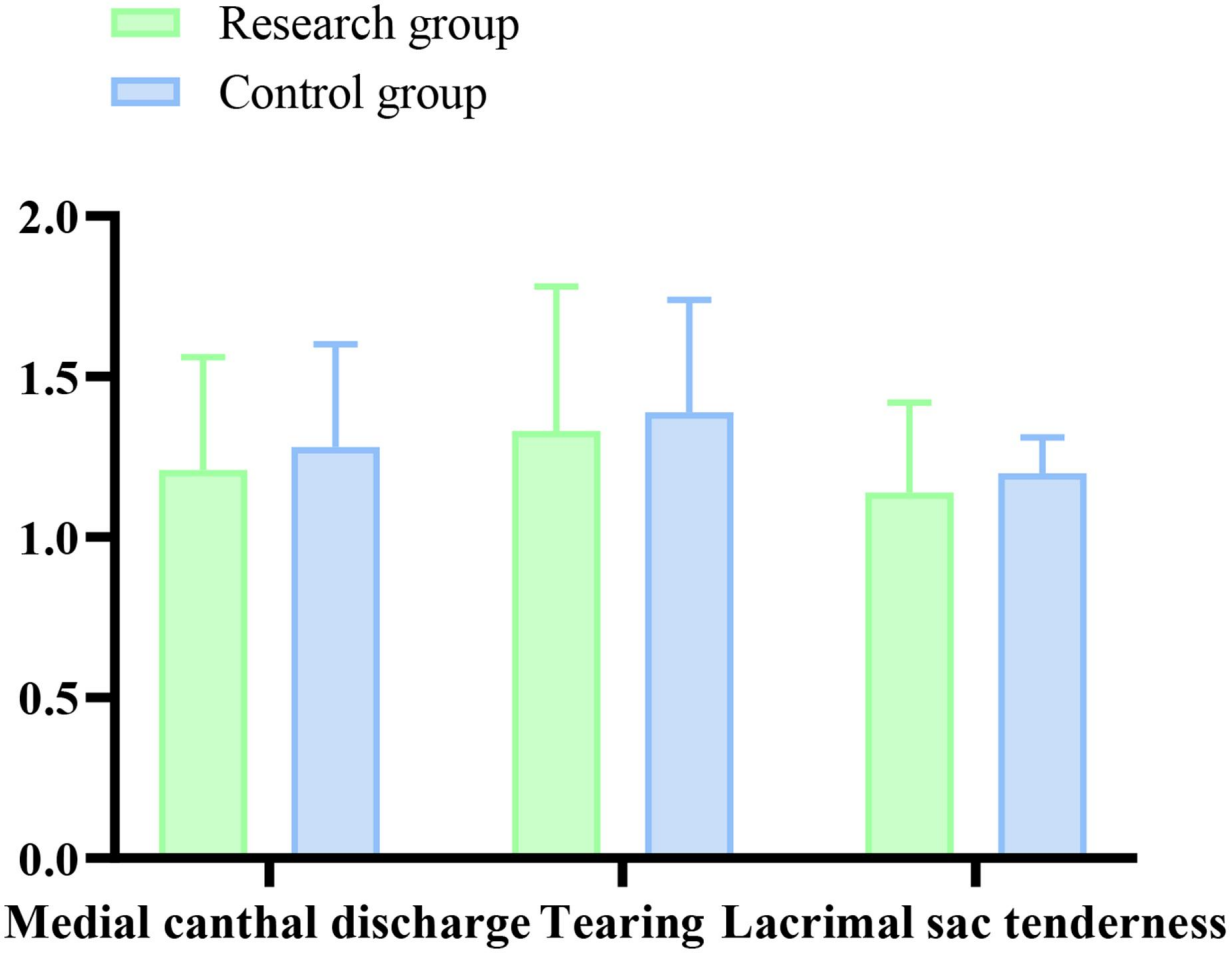
Comparison of main symptom scores between the two groups. The figure provides a descriptive visualization of symptom scores. Between-group statistical comparisons were performed using the Mann–Whitney U test because the symptom scores were ordinal variables.

### 3.4 Clinical indicators

The mean surgical duration was 37.25 ± 5.41 min in the research group and 28.98 ± 5.11 min in the control group, corresponding to a mean difference of 8.27 min (95% CI: 4.90–11.64; *P* < 0.001). In contrast, intraoperative blood loss was markedly lower in the research group than in the control group (26.58 ± 4.73 vs. 125.23 ± 8.11 mL; mean difference=−98.65 mL, 95% CI: −102.90 to −94.40; *P* < 0.001). Postoperative hospital stay was also shorter in the research group (3.71 ± 1.11 vs. 6.01 ± 2.25 days; mean difference=−2.30 days, 95% CI: −3.44 to −1.16; *P* < 0.001), as shown in Figure 3.

**Figure 3:**
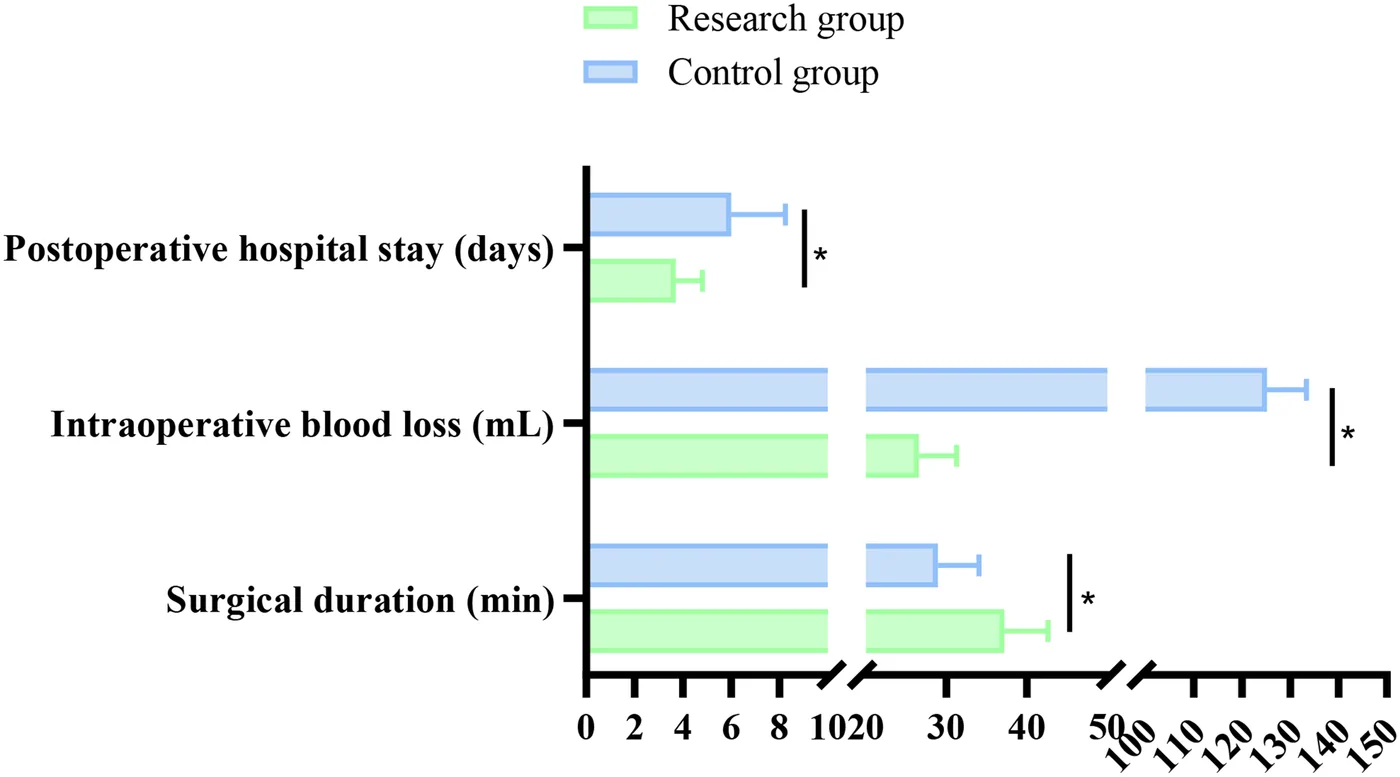
Comparison of clinical indicators between the two groups. * indicates a statistically significant difference between the two groups (*P* < 0.05).

### 3.5 Adverse reactions

In the research group, 1 of 20 patients (5.0%) experienced a postoperative adverse event, consisting of one case of hemorrhage (1/20, 5.0%), with no cases of infection or other documented complications. In the control group, 9 of 20 patients (45.0%) experienced postoperative adverse events, including 3 cases of infection (3/20, 15.0%), 4 cases of hemorrhage (4/20, 20.0%), and 2 cases classified as other documented complications (2/20, 10.0%). The between-group differences were not statistically significant for infection (Fisher's exact *P* = 0.231), hemorrhage (*P* = 0.342), or other complications (*P* = 0.487) when evaluated separately. However, the overall incidence of postoperative adverse events was significantly lower in the research group than in the control group [1/20 [5.0%] vs. 9/20 [45.0%]; Fisher's exact *P* = 0.008; OR = 0.064, 95% CI: 0.007–0.578], as shown in Figure 4.

**Figure 4:**
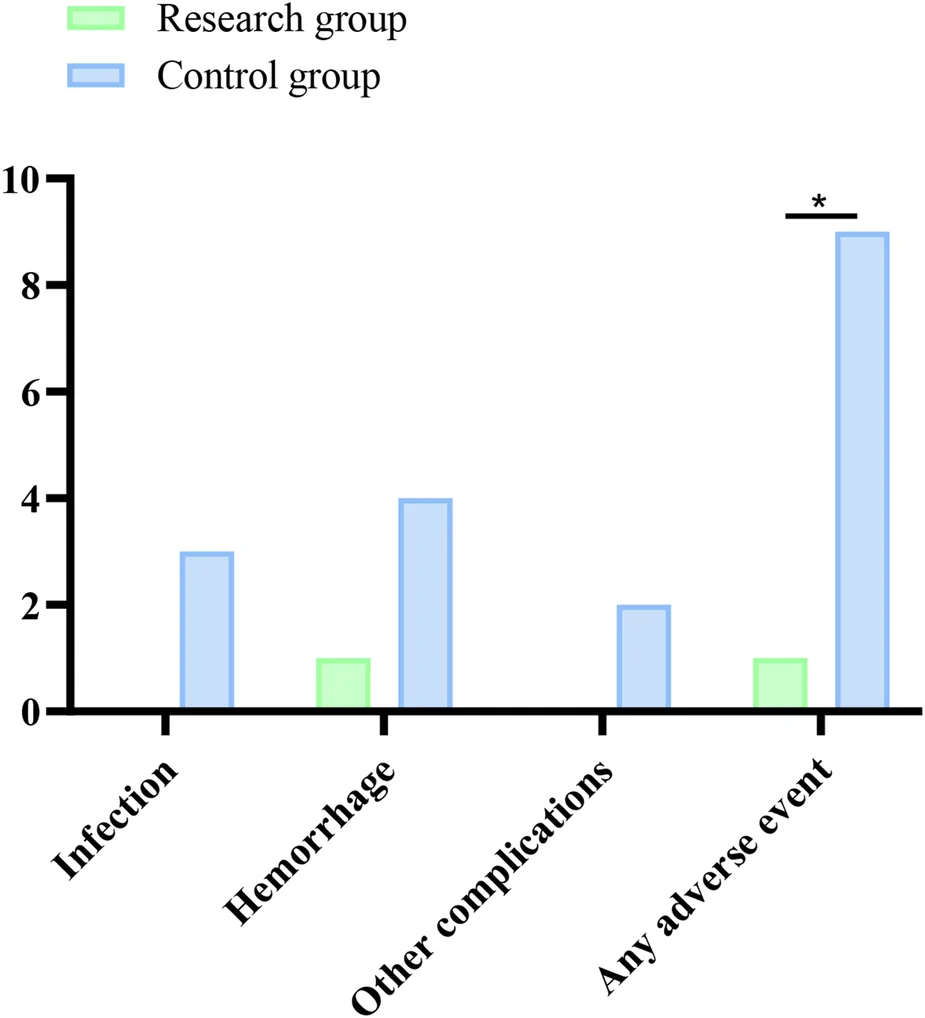
Comparison of postoperative adverse events between the two groups. * indicates a statistically significant between-group difference in the overall adverse-event incidence (Fisher's exact *P* = 0.008).

### 3.6 Patient-reported satisfaction

At the 2-month follow-up, 8 of 20 patients (40.0%) in the research group reported being very satisfied, 11 (55.0%) reported being satisfied, and 1 (5.0%) reported being dissatisfied. In the control group, 4 of 20 patients (20.0%) reported being very satisfied, 8 (40.0%) reported being satisfied, and 8 (40.0%) reported being dissatisfied. The overall self-reported satisfaction rate was therefore 95.0% (19/20) in the research group and 60.0% (12/20) in the control group. Overall self-reported satisfaction was higher in the research group (Fisher's exact *P* = 0.020; OR = 12.67, 95% CI: 1.40–114.42), as shown in Figure 5.

**Figure 5:**
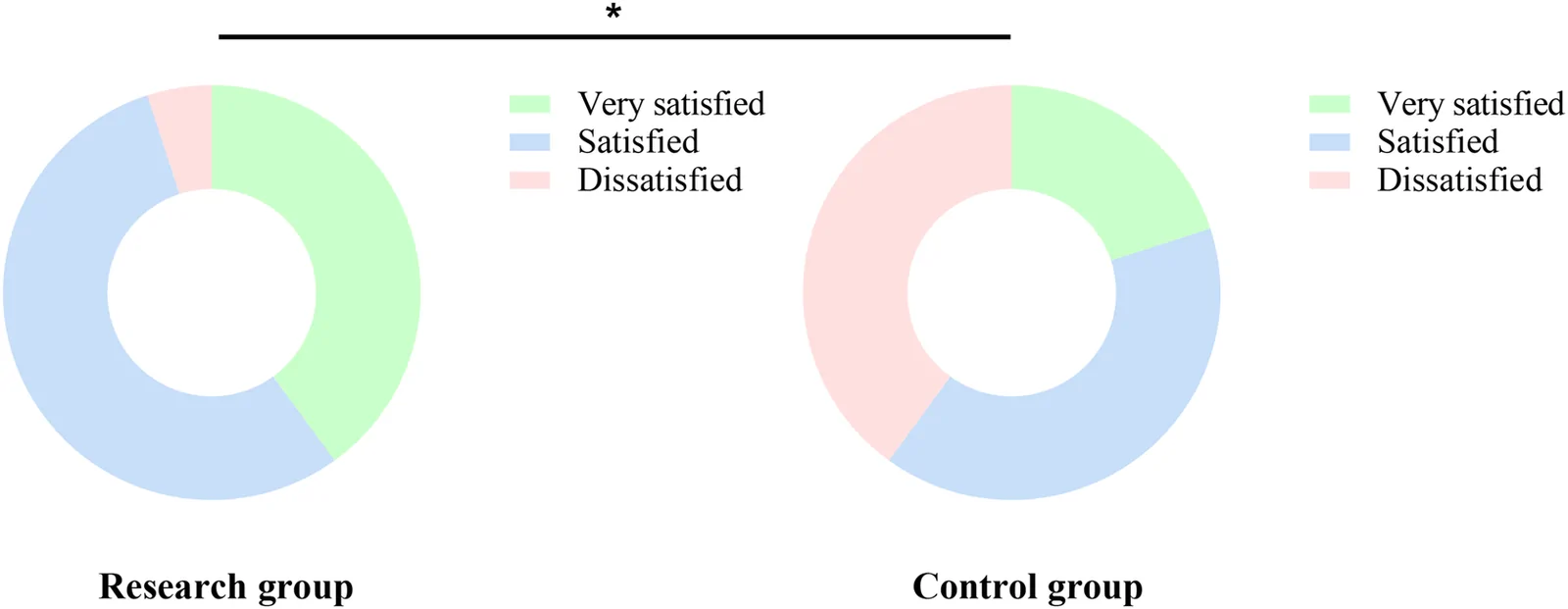
Comparison of patient-reported satisfaction between the two groups. * indicates a statistically significant difference between the two groups, *P* < 0.05. Satisfaction was assessed using a study-specific, non-validated three-category patient self-report questionnaire.

## 4 Discussion

Chronic dacryocystitis is a common ophthalmic disease closely related to factors such as age, gender, occupation, and living environment, predominantly affecting middle-aged and elderly women. The primary cause is nasolacrimal duct obstruction or stenosis, leading to the retention of secretions in the lacrimal sac, which subsequently results in inflammation and infection (8, 9). The prolonged accumulation of purulent fluid in the lacrimal sac provides an ideal environment for bacterial proliferation. This condition, compounded by increased secretions that cannot be drained, may eventually lead to sac expansion or cyst formation (10, 11). Chronic untreated dacryocystitis can lead to severe complications, including inflammatory infections and systemic bacterial infections. If not promptly managed, the reflux of purulent discharge may cause corneal ulcers or refractory keratitis through corneal lesions, potentially resulting in severe outcomes such as endophthalmitis. Therefore, resolving dacryocystitis completely before intraocular surgery is a critical step in preventing postoperative infections (12–14).

The traditional external approach to dacryocystorhinostomy is a classic method for treating chronic dacryocystitis. However, it involves a larger surgical incision, noticeable postoperative scarring, and a higher rate of complications. With advances in medical technology, endoscopic endonasal dacryocystorhinostomy has been increasingly applied in clinical practice. This technique preserves the functional structures of the nasal cavity and lacrimal duct, offering significant advantages such as minimal trauma, a clear intraoperative field of vision, and faster postoperative recovery. Additionally, endoscopic techniques allow for the simultaneous treatment of related nasal conditions, such as nasal polyps and deviated nasal septum, thereby improving overall therapeutic efficacy (15–18). Through retrospective analysis, this study systematically compared the clinical efficacy and safety of traditional external dacryocystorhinostomy with endoscopic endonasal dacryocystorhinostomy in treating chronic dacryocystitis, providing exploratory comparative data on the short-term outcomes of the two surgical approaches.

Previous studies have reported no statistically significant differences in treatment efficacy between endoscopic endonasal dacryocystorhinostomy (EEDCR) and traditional external dacryocystorhinostomy (EXT-DCR) (19–21). In the present study, no statistically significant difference in short-term clinical efficacy was detected between the two groups. Separate assessment of anatomical and functional success at 2 months likewise showed no statistically significant between-group differences, suggesting broadly similar short-term patterns within the available sample while not establishing equivalence between the two procedures. However, given the limited sample size, this finding should not be interpreted as demonstrating equivalence or non-inferiority between the two surgical approaches. Rather, it indicates that the present study did not detect a between-group difference within the available sample. Previous reports have also described favorable short-term outcomes following both procedures. These observations are generally consistent with the present findings, but larger adequately powered studies are required to determine whether clinically meaningful differences in efficacy exist between EEDCR and EXT-DCR.

In the unadjusted comparisons, the EEDCR group had a longer surgical duration but lower intraoperative blood loss, a shorter postoperative hospital stay, a lower overall incidence of postoperative adverse events, and higher self-reported satisfaction than the EXT-DCR group. These findings suggest potential short-term perioperative advantages of EEDCR in selected outcomes; however, they should not be interpreted as evidence of overall superiority because treatment allocation was non-random and potential confounding factors were not adjusted for. Firstly, regarding intraoperative blood loss, the direct visualization offered by EEDCR allows precise tissue dissection while avoiding critical vascular structures, significantly reducing surgical bleeding. In contrast, the traditional external approach involves multiple layers of skin and soft tissue, resulting in greater surgical trauma and a higher risk of bleeding. Secondly, patients in the EEDCR group had a significantly shorter postoperative hospital stay compared to the control group. This is likely due to the minimal invasiveness and faster recovery associated with the endoscopic technique. Early discharge not only reduces the financial burden on patients but also minimizes the risk of hospital-acquired infections (22–25). The overall incidence of postoperative adverse events was significantly lower in the EEDCR group than in the EXT-DCR group. Studies suggest that the excellent anatomical exposure provided by endoscopy effectively prevents intraoperative damage to critical structures and reduces the likelihood of severe complications. Additionally, the absence of facial scars in EEDCR contributes to improved postoperative comfort and patient satisfaction, as this technique does not require an external incision, thus eliminating the risk of scar formation (26–28).

Finally, regarding patient satisfaction, the EEDCR group demonstrated significantly higher satisfaction compared to the traditional EXT-DCR group. The higher self-reported satisfaction observed in the EEDCR group may be related to patients’ perceptions of postoperative recovery and cosmetic outcomes; however, this finding should be interpreted cautiously because satisfaction was assessed using a non-validated self-report instrument and patients were aware of the surgical approach received. Compared to traditional techniques, the minimally invasive nature of EEDCR aligns more closely with modern patients’ demands for treatment safety and aesthetic appeal. In summary, endoscopic endonasal dacryocystorhinostomy has been widely applied in clinical practice. Unlike traditional external approaches, this technique avoids damage to the medial canthal ligament and orbicularis oculi muscle, preserving the lacrimal pump function, enabling faster postoperative recovery, and leaving no skin scars, making it more acceptable to patients. Endoscopic visualization may facilitate precise localization of the lacrimal sac and adjacent nasal structures; however, the surgical duration was longer in the EEDCR group in the present study. Therefore, the current findings do not support an advantage of EEDCR in terms of operative time. However, the safety of the procedure relies on the surgeon's comprehensive understanding of the anatomy of the lacrimal sac, nasal cavity, and the entire head and facial structure. Without this expertise, complications such as orbital wall fractures, extraocular muscle injuries, and optic nerve damage may occur (29–31).

Within the 2-month follow-up period of the present study, EEDCR showed favorable short-term perioperative, safety, and patient-reported outcomes compared with EXT-DCR. However, these findings do not establish the durability of treatment success beyond the short follow-up period. Despite the significant clinical advantages of EEDCR, this study has some limitations. First, as a single-center retrospective study with a relatively small sample size, the results may have limited generalizability. The limited sample size also reduced statistical power, particularly for detecting modest differences in clinical efficacy and relatively infrequent adverse events. Therefore, non-significant findings should not be interpreted as evidence of equivalence between the two procedures. In addition, the surgical approach was not randomly assigned but was determined during routine clinical care, and therefore selection bias and residual confounding related to surgical decision-making cannot be excluded. Furthermore, no multivariable or propensity-score adjustment was performed because of the limited sample size and number of outcome events. Therefore, the observed between-group associations may have been influenced by measured or unmeasured confounding, and no independent treatment effect can be inferred from the present analysis. Third, patient satisfaction was assessed using a study-specific, non-validated self-report questionnaire. No independent blinded assessor was involved, and patients could not be blinded to the surgical approach because the presence or absence of an external facial incision made the two procedures readily distinguishable. Therefore, the observed difference in satisfaction should be interpreted cautiously because it may have been influenced by response bias, expectations regarding cosmetic outcomes, and awareness of the surgical approach. Second, the 2-month postoperative follow-up was insufficient to determine the durability of lacrimal drainage patency or to adequately assess recurrence and late postoperative complications. Therefore, the present findings should be interpreted as short-term outcomes rather than evidence of sustained long-term efficacy or safety. Longer follow-up is required to evaluate the durability of the observed results. Future research should focus on multicenter, large-sample, prospective studies to further validate the conclusions of this study and explore additional optimization strategies.

## 5 Conclusion

In this small retrospective study, unadjusted comparisons within the 2-month postoperative follow-up showed that endoscopic endonasal DCR was associated with lower intraoperative blood loss, shorter postoperative hospital stay, a lower overall incidence of postoperative adverse events, and higher self-reported patient satisfaction than traditional external DCR, although surgical duration was longer. Short-term anatomical and functional success rates did not differ significantly between the two groups. Given the retrospective design, small sample size, absence of adjustment for potential confounders, and limited follow-up duration, these findings should be regarded as exploratory short-term comparative results and should not be interpreted as establishing EEDCR as the preferred surgical approach. Larger prospective studies with adequate adjustment for confounding and longer follow-up are needed to confirm these findings.

## Data Availability

The original contributions presented in the study are included in the article/Supplementary Material, further inquiries can be directed to the corresponding author.
